# Folate and colorectal cancer prevention

**DOI:** 10.1038/sj.bjc.6604823

**Published:** 2008-12-16

**Authors:** R A Hubner, R S Houlston

**Affiliations:** 1 Gastrointestinal Unit, Royal Marsden Hospital, Fulham Road, London, UK; 2 Section of Cancer Genetics, Institute of Cancer Research, Surrey, UK

**Keywords:** folate, colorectal cancer, prevention

## Abstract

Anti-folate chemotherapy agents such as methotrexate and fluorouracil reduce proliferation of neoplastic cells by inhibiting DNA synthesis. Paradoxically epidemiological data suggests an inverse relationship between dietary folate intake and incidence of colorectal cancer (CRC). On the basis of this and other putative health benefits around 35% of the North American population take folic acid supplements, in addition to natural food folates and fortified flour and cereal grains. Recently, randomised controlled trials investigating folic acid as a secondary preventative agent in colorectal neoplasia have shed further light on the relationship between folate and colorectal carcinogenesis, corroborating data from animal models indicating opposing effects dependent on the timing of exposure in relation to the development of neoplastic foci. A ‘dual-modulator’ role for folate in colorectal carcinogenesis has been proposed in which moderate dietary increases initiated before the establishment of neoplastic foci have a protective influence, whereas excessive intake or increased intake once early lesions are established increases tumorigenesis. Functional polymorphic variants in genes encoding key enzymes in the folate metabolic pathway add a further layer of complexity to the relationship between folate and CRC risk. Here, we review the evidence concerning the efficacy and safety of folate as a potential CRC chemopreventive agent.

In the United States and United Kingdom colorectal cancer (CRC) is respectively the second and third most common malignancy (Jemal *et al*, 2007). Despite new therapeutic agents the prognosis from CRC remains relatively poor, and the median survival of optimally treated patients with advanced disease is ∼20 months. Thus there is an urgent need to identify agents with CRC chemopreventive activity to reduce the burden of this disease on both the individual and wider society.

Folate is a natural constituent of foods such as green leafy vegetables, legumes, and citrus fruits, and has attracted considerable attention as a potential CRC chemopreventive agent. Here, we review our current understanding of the relationship between folate and colorectal neoplasia development.

## Folate metabolism, DNA methylation and dna synthesis

Folate is a generic term for a naturally occurring family of B-group vitamins composed of an aromatic pteridine ring linked to *p*-aminobenzoic acid and a glutamate residue (Shane, 1995). Following absorption in the small intestine dietary food folates undergo hydrolysis to methyltetrahydrofolate (methylTHF), the predominant form of folate in plasma (Shane, 1995). Folic acid also undergoes conversion to methylTHF, but this process becomes saturated at doses of ∼270 *μ*g and at higher levels folic acid is transported directly into the plasma (Sweeney *et al*, 2007). Hence, daily ingestion of 400 *μ*g, a dose commonly used in supplements, produces a sustained level of plasma folic acid.

**Figure 1:**
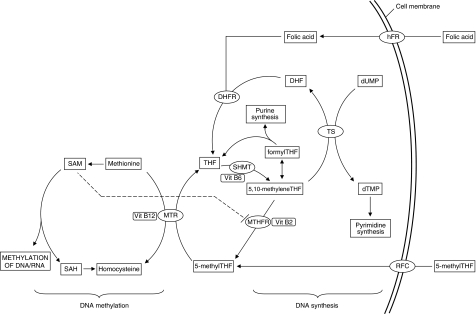
Schematic representation of folate metabolism illustrating the entry of natural folates and folic acid into the pathway, and flow of methyl groups towards either DNA synthesis or DNA methylation. RFC, reduced folate carrier; hFR, human folate receptor; MTR, methionine synthase; MTHFR, methylenetetrahydrofolate reductase; SHMT, serine hydroxymethlytransferase; TS, thymidylate synthase; THF, tetrahydrofolate; DHF, dihydrofolate; SAM, *S*-adenosylmethionine; SAH, *S*-adenosylhomocysteine; dUMP, deoxyuridine monophosphate; dTMP, deoxythymidine monophosphate.

Cellular folates act as donors and acceptors of methyl groups in the biosynthesis of nucleotide precursors used for DNA synthesis, and provision of methyl groups for methylation of DNA, RNA, and proteins (Figure 1; Shane, 1995). These important cellular processes lie at opposite ends of folate metabolism linked by methylenetetrahydrofolate reductase (MTHFR) which catalyses the irreversible conversion of 5,10-methyleneTHF to 5-methylTHF. The MTHFR substrate, 5,10-methyleneTHF, is also a substrate for the thymidylate synthase (TS) enzyme in the methylation of deoxyuridine monophosphate (dUMP) to deoxythymidine monophosphate (dTMP), which is the sole *de novo* source of thymidine and the rate limiting step in DNA synthesis in mammalian cells (Choi and Mason, 2002). 5,10-methyleneTHF is also used in the production of formylTHF, which is in turn used in *de novo* purine synthesis. The MTHFR product, 5-methylTHF, is the methyl group donor for the remethylation of homocysteine to methionine catalysed by the enzyme methionine synthase (MTR). Methionine is adenylated to form *S*-adenosylmethionine (SAM), which is the methyl group donor in methylation reactions, whereas SAM inhibits the MTHFR enzyme providing a negative feedback control loop.

Variation in the distribution of methyl groups through altered folate metabolism impacts on both DNA methylation and DNA synthesis, two crucial cellular processes in relation to neoplastic transformation (Choi and Mason, 2002). Methylation of the cytosine residues of cytosine–guanine dinucleotide pairs is an important epigenetic determinant of gene expression and also has a role in maintaining DNA stability. Hypermethylation of gene promoter regions results in loss of tumour suppressor gene function, whereas reduced ‘global’ methylation results in chromosomal instability and an increase in mutational events (Eden *et al*, 2003). Long-term dietary folate deficiency in humans results in global DNA hypomethylation in lymphocytes, which is reversible on repletion of folate status (Jacob *et al*, 1998). Paradoxically studies in rodents have shown promoter-specific hypermethylation increases in response to folate deficiency (Pogribny *et al*, 1997). Through a reduction in the availability of methyl groups for methylation of homocysteine to methionine, folate deficiency leads to a rise in homocysteine levels which in turn results in raised levels of *S*-adenosylhomocysteine (SAH). *S*-adenosylhomocysteine is an inhibitor of methylation reactions, and this is thought to underlie the reduced global DNA methylation observed in individuals with low folate status.

Reduced deoxythymidylate synthesis leading to uracil misincorporation during DNA synthesis provides a second mechanism through which folate deficiency can disrupt DNA integrity and promote carcinogenesis. Removal of a uracil base involves creation of a single-strand DNA break, and where two adjacent uracil bases lie on opposite strands a double-strand DNA break will occur which are difficult to repair and are associated with an increased cancer risk (McKinnon and Caldecott, 2007). Increased DNA uracil content and chromosomal breaks occur in folate-deficient humans, and both can be reversed by restoration of adequate folate status (Blount *et al*, 1997).

The understanding that folate metabolism can reciprocally influence both DNA synthesis and methylation has made environmental and genetic variants that impact on this pathway attractive candidates as cancer susceptibility factors. These include dietary intakes of folate and folic acid, and functional polymorphisms in the genes coding for folate metabolism enzymes.

## Epidemiological studies of folate intake and colorectal cancer risk

Thirteen case–control studies and eight cohort studies have examined the relationship between folate intake and CRC (Giovannucci, 2002) with most reporting reductions in cancer incidence in individuals with higher folate intake. A meta-analysis of seven of the eight cohort studies indicated a 25% reduction in CRC risk for subjects in the highest quintile of dietary folate intake (from food only) compared with the lowest (Sanjoaquin *et al*, 2005). More recent studies, however, have not confirmed an inverse relationship even in subjects with a relatively low mean dietary folate intake (de Vogel *et al*, 2008, Sharp *et al*, 2008). Subgroup analyses in the study by Sanjoaquin *et al*. (2005) indicated a stronger protective effect for colon compared with rectal cancer, whereas when total folate intake (food and supplements combined) was examined there was no statistically significant evidence for a protective effect of high intake for either colon of rectal cancers. Further studies are required to clarify whether this discrepancy between the effects of dietary and total folate intake is genuine, but the finding might be a consequence of greater confounding by other dietary factors when analysing the association between CRC and dietary folate than that with total folate. Alternatively, natural folates and synthetic folic acid may have different impacts on the folate metabolic pathway.

It is likely that folic acid supplements must be taken for a sustained period to impact on CRC risk. Such an assertion is supported by findings from the Nurses’ Health Study, a cohort study with over 88 000 female participants, which showed no inverse association between CRC risk and supplementation when taken for less than 5 years, a non-significant inverse association when taken for 5–14 years, but a substantial and significant 75% risk reduction when taken for 15 years or more (Giovannucci *et al*, 1998). A similarly long latency period between folic acid supplementation and a reduction in risk of CRC was demonstrated in an independent large cohort study (Jacobs *et al*, 2003), in which regular supplement use at enrolment had no association with CRC incidence whereas supplement use 10-years before enrolment was significantly inversely associated.

Retrospective (Boyapati *et al*, 2004) and prospective (Giovannucci *et al*, 1993) studies examining folate intake and risk of colorectal adenoma (CRA), the major precursor lesion for most CRC, also provide support for an inverse relationship between folate exposure and colorectal neoplasia risk. In an analysis of two cohort studies, the Nurses’ Health study and the Health Physicians Follow-Up Study, significantly reduced risks were observed in both women and men for individuals in the highest quintile of total folate intake compared with the lowest (Giovannucci *et al*, 1993).

Difficulties in accurately estimating dietary folate intake have prompted some investigators to analyse the relationship between CRC risk and red blood cell or plasma folate levels. These variables may provide a more accurate measure of folate status at the expense of being more susceptible to short-term fluctuations in dietary folate intake. A nested case–control study based on the Women's Health Study cohort reported a significant 50% reduction in CRC risk in women in the highest quartile of plasma folate compared with the lowest (Kato *et al*, 1999), and a similar CRC risk reduction was observed in male participants with adequate plasma folate levels compared to those with levels indicating deficiency (<3 ng ml^−1^) in the Physicians Health Study, albeit non-significantly (Ma *et al*, 1997). However, more recent studies have not supported an inverse association between plasma folate and CRC risk (Van Guelpen *et al*, 2006, Otani *et al*, 2008). A nested case–control study in the Northern Sweden Health and Disease Cohort reported that subjects with over 4 years follow-up and plasma folate levels in the highest quintile were at a 4-fold increased risk of CRC compared with those in the lowest quintile (Van Guelpen *et al*, 2006), whereas a Japanese cohort study including 375 individuals diagnosed with CRC provided no evidence of a relationship between plasma folate and CRC risk in either men or women (Otani *et al*, 2008).

Other dietary factors apart from folate and folic acid may also impact on folate metabolism, notably alcohol, choline, and methionine. Alcohol is a folate antagonist interfering with folate absorption and other aspects of folate metabolism, whereas choline and methionine are important dietary sources of methyl groups, estimated to contribute respectively 60 and 20% of the total ingested by humans, compared with 20% derived from folate. Diets high in alcohol and low in folate and methionine are considered ‘methyl-poor’ whereas those low in alcohol and high in folate and methionine are ‘methyl-rich’. Studies that have analysed combinations of these dietary components have generally found increased risks of CRC and CRA for ‘methyl-poor’ diets compared with ‘methyl-rich’ providing further evidence of a protective role for efficient folate metabolism in colorectal neoplasia (Giovannucci, 2002).

Collectively, the evidence from epidemiological studies is at best only suggestive of an inverse relationship between dietary folate or supplemental folic acid intake and CRC risk. Important questions remain unresolved, however, including the optimal level of increased dietary or supplemental folate intake to minimise CRC risk, the duration of increased exposure required, whether cancer risks are attenuated in both men and women and in both the colon and rectum, and whether dietary folates and supplemental folic acid have similar inverse associations. It is notable that the recent World Cancer Research Fund/American Institute for Cancer Research report into food, nutrition, physical activity and the prevention of cancer judged foods containing folate to have only ‘limited-suggestive’ evidence of a protective effect against CRC (WCRF/AICR, 2007).

## Functional polymorphisms in folate metabolism genes

A number of common genetic variants alter either the cellular levels or functioning of folate metabolism enzymes, and are likely to play an important role in determining an individual's response to changes in dietary folate intake. The C677T variant of the *MTHFR* gene has been extensively investigated for an association with CRC and CRA risk and interactions with intakes of folate, other B vitamins, and alcohol. Individuals with homozygous 677TT genotype, who comprise ∼11% of Caucasians, have only 35% of the normal activity of this pivotal enzyme, resulting in channeling of methyl groups towards DNA synthesis at the expense of DNA methylation (Chen *et al*, 1996). When genotype is considered in isolation, 677TT individuals show a modest reduction in CRC risk compared to those with wild-type 677CC genotype (Hubner and Houlston, 2007), whereas CRA risk does not appear to be significantly influenced by *MTHFR* C677T genotype (Sharp and Little, 2004). Studies stratifying by both genotype and methyl status, however, have indicated a more complex relationship with 677TT individuals being at reduced CRC risk if they also have high methyl status (low alcohol, high folate), but at increased risk if their methyl status is low (high alcohol, low folate) (Chen *et al*, 1996; Ma *et al*, 1997). These interactions are not universally reported in small case–control series, although a recent well-powered case–control study also indicated interactions between *MTHFR* C677T genotype and folate status or alcohol intake in determining CRC risk (Le Marchand *et al*, 2005).

The complex relationship between folate intake and CRC risk may be further modulated by other functional polymorphisms impacting on folate metabolism. These include the *MTHFR* A1298C variant which also confers a reduced MTHFR enzyme function, the thymidylate synthase promoter (*TSER*) and the 3′ untranslated region (*TS* 1494del6) variant (Sharp and Little, 2004; Ulrich *et al*, 2005). Furthermore, recent data indicates folate may have differential influences on the development of CRC caused by mis-match repair deficiency and chromosomal instability (Hubner *et al*, 2007).

To date, studies investigating the relationships between the multiple genetic and dietary factors that impact on folate metabolism have had sample sizes at least one order of magnitude too small to convincingly confirm or refute potential gene–gene or gene–nutrient interactions in determining colorectal neoplasia risk. It is highly probable, however, that studies seeking to define the relationship between folate intake and CRC risk that do not stratify subjects by genotype at two or three polymorphic loci at the very least will miss important disease–nutrient associations in subgroups of the population defined by these genetic variants.

## Evidence from animal models

The relationship between folate and intestinal neoplasia has been investigated with two classes of rodent model; animals injected with chemical colorectal carcinogens such as dimethylhydrazine or azoxymethane, and animals genetically predisposed to intestinal neoplasia (Cravo *et al*, 1992; Kim *et al*, 1996; Song *et al*, 2000a, 2000b). Studies using chemical carcinogens reported that moderate folate deficiency enhanced colorectal carcinogenesis whereas folate supplementation had an opposing effect (Cravo *et al*, 1992; Kim *et al*, 1996). Levels of folate supplementation over four times the basal daily requirement did not confer additional benefit however, and exceptionally high levels of folate supplementation (up to 1000 times basal requirement), were found to promote the development of aberrant crypt foci and early neoplasms (Kim *et al*, 1996).

In *APC*^Min^ mice randomly assigned to receive diets of varying folate content after weaning, the number of small intestinal adenomas found at 3 months was inversely related to both the level of dietary folate and serum levels of folate, again corroborating evidence from humans (Song *et al*, 2000a). At 6 months however, mice fed a folate-deficient diet had fewer distal small intestinal adenomas compared with folate-supplemented animals, and the number of distal adenomas was positively associated with serum folate levels suggesting an inhibitory effect of folate deficiency on established distal small intestinal adenomas (Song *et al*, 2000a). A similar study using *APC*^+/−^*MSH2*^−/−^ mice also showed differential associations of dietary folate depending on the timing of intervention (Song *et al*, 2000b). When commenced before the establishment of neoplastic foci, folate supplementation at four times basal requirement resulted in a 3-fold reduction in the number of small intestinal adenomas compared with a moderately folate-deficient diet. Dietary folate manipulation after the development of neoplastic foci had the opposite effect however, with deficiency significantly decreasing the number of adenoma compared with supplementation.

Histological, clinical, and molecular genetic differences prevent direct extrapolation of the findings from both types of animal models to human CRC. For example, the carcinomas developing in the chemical carcinogen models often arise from flat foci of dysplasia rather than adenomas, whilst in both *APC*^Min^ and *APC*^+/−^*MSH2*^−/−^ mice the predominant phenotype is small intestinal rather than colonic adenomas and animals do not develop adenocarcinomas as they die from florid polyposis and intestinal obstruction (Song *et al*, 2000a, 2000b). In genetic models, the aberrant cypt foci (ACF) to adenoma progression typical of sporadic human CRA is not established, and *KRAS* mutations commonly observed in human CRCs are not detected in *APC*^Min^ mice polyps (Song *et al*, 2000a). Despite such limitations, these studies provide further evidence that the relationship between dietary folate intake and intestinal neoplasia is not straightforward with both the level and timing of exposure being important determinants. These observations have played an important role in the development of a hypothesis proposing a ‘dual-modulator’ role for folate in CRC, with a protective influence of moderate dietary increases initiated before the establishment of neoplastic foci, but a detrimental influence of excessive intake or increased intake in the setting of pre-established neoplastic foci (Kim, 2004; Ulrich and Potter, 2007). Early prevention of tumorigenesis is most likely because of protection against DNA damage by maintaining adequate methyl groups for DNA methylation and for purine and pyrimidine synthesis, whereas the probable mechanism underlying the later enhanced tumour growth is an increased provision of nucleotide precursors to rapidly replicating neoplastic cells allowing accelerated proliferation and growth.

## Randomised studies of folic acid intervention in humans

Randomised controlled trials of potential CRC preventive agents in the primary preventive setting would require a prohibitively large number of participants because of the long latent period and relatively low population incidence of the disease. For this reason a number of dietary and other interventions have been investigated in the secondary preventive setting of CRA recurrence. The CRA recurrence paradigm has provided evidence of chemopreventive activity for calcium supplements, aspirin, and COX-2 selective NSAIDs, and an absence of activity for wheat bran high-fibre cereal supplement, ursodeoxycholic acid, and a low-fat high-fibre high-fruit diet.

Very recently the results of two large, multi-centre, phase III studies – The Aspirin/Folate Polyp Prevention study and the United Kingdom Colorectal Adenoma Prevention study – have been published (Table 1; Cole *et al*, 2007; Logan *et al*, 2008). Both studies used a ‘factorial’ design in which participants were not only randomised to folic acid and placebo, but also aspirin and placebo, allowing the effects of both agents to be investigated simultaneously. The US-based study reported by Cole *et al* (2007) randomised individuals to 1000 *μ*g folic acid daily for 3 years, and recurrence data were available for 987 individuals. The incidence of at least one CRA, the primary end point of the study, was essentially the same in the folic acid and placebo arms; however, subjects receiving folic acid showed non-significant increases of 32 and 20%, respectively in the secondary end points of incidence of advanced CRA and the number of subjects with ⩾3 CRA. Participants were invited to continue in the trial for a further 3–5 years, and those that agreed could either continue study medication in their original randomised group or discontinue medication and simply be followed-up for further end points. At six to eight years of follow-up 607 individuals remained for analysis of recurrence data, 501 of whom had continued randomised medication. In this second follow-up interval individuals in the folic acid arm showed a non-significant 13% increase in the incidence of at least one CRA recurrence, a 63% increase in advanced CRA, and the number of subjects with three or more CRA was more than doubled. In participants with end point information for both follow-up intervals the adenoma rate (any CRA in either interval) was 65.5% in the placebo group and 71.3% in the folic acid group (RR=1.09; 95% CI: 0.98–1.21). These results indicated that a daily dose of 1000 *μ*g folic acid did not result in a reduction in CRA recurrence; however, the more concerning conclusion was a possible increased risk of advanced CRA or multiple CRA. A plausible explanation for these unexpected findings is that folic acid promoted the growth of pre-existing ACF or microscopic adenoma that were not visible at entry colonoscopy, in a similar manner to that observed in the genetic models discussed above (Ulrich and Potter, 2007). Of further concern was a significant increase in cancers other than CRC, largely attributable to an elevated risk of prostate cancer (Cole *et al*, 2007).

The second large RCT of folic acid in CRA recurrence also failed to show an inverse association, but did not provide confirmatory evidence of a positive association (Logan *et al*, 2008). This UK-based study randomised individuals to 500 *μ*g daily folic acid or placebo for 3 years, used similar end points, and reported recurrence data for 853 participants. Subjects who received folic acid had only a very minor increase in incidence of one or more adenoma, and no increase in advanced adenoma. Longer term follow-up data are not available from this trial. Possible reasons for the discrepancies with the findings of the US trial are the lower dose of folic acid used in the UK trial, and a likely lower dietary intake of folate because of the absence of mandatory folate fortification of flour in the United Kingdom.

Although inconclusive, the results from CRA recurrence studies suggest that folic acid supplementation in individuals at increased risk of CRC on the basis of previous or existing CRA is unlikely to be beneficial for CRC prevention, and could possibly be detrimental, particularly at high doses. As 25–50% of North Americans over the age of 50 harbour CRAs even only a minor increase in risk could translate to a significant public health concern. The absence of a clear association between *MTHFR* C677T genotype and CRA incidence may make interpretation of associations between folic acid intake and CRA recurrence risk in relation to CRC risk more complex, although there is evidence that *MTHFR* polymorphism genotypes influence CRA recurrence in a similar manner to CRC incidence (Martinez *et al*, 2006).

## Folate fortification and CRC incidence

Folic acid supplements taken in the peri-conceptual period have proven benefits in reducing neural tube defects (NTDs; MRC Vitamin Study Research Group, 1991). In March 1996, the US Food and Drug Administration issued a mandate requiring fortification of flour with folic acid (140 *μ*g folic acid per 100 g flour) with the aim of reducing NTDs. Although the mandate specified that fortification should be in place by no later than January 1998, in practice most large food companies commenced fortification in early 1996. Fortification was also mandated in Canada although was not allowed to commence before December 1996. Mandatory folic acid fortification has been highly successful in achieving its primary aim with reductions in the incidence of NTDs in North America of 20–50%. The typical intakes of folic acid from fortified foods were more than two times the level originally predicted however, and population-based studies indicated that plasma folate levels in adults increased ∼2-fold following fortification.

**Figure 2:**
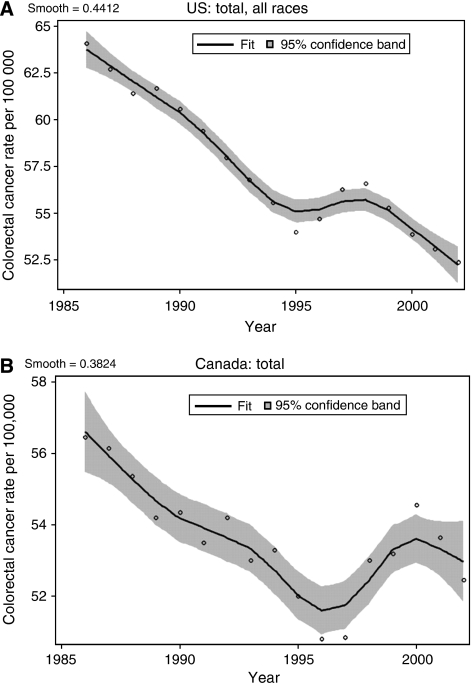
Age-adjusted CRC incidence from 1986 to 2002 in the United States (**A**) and Canada (**B**) based on nationally representative databases (for Canada, the incidence is based on the average of the rates for men and women). Open circles represent data points. A nonparametric loess smoother was fitted to the data and the grey areas represent 95% confidence bands. (Reproduced by the kind permission of the American Association of Cancer Research from Mason *et al*, 2007).

A recent study has highlighted a temporal relationship between the onset of folic acid fortification and rises in the incidence of CRC in both the United States and Canada (Mason *et al*, 2007). The analyses by Mason *et al* (2007) demonstrate that in the early 1990s the age-adjusted incidence of CRC declined moderately in both countries (Figure 2). Between 1995 and 1996 however, the incidence rate in the United States showed a slight increase followed by more marked increases in 1997 and 1998. A similar change occurred in Canada, but with a time lag of ∼1 year compared to the United States, with a sharp increase occurring between 1997 and 1998. In both instances the deviations in CRC incidences from the pre-existing trends were highly statistically significant, occurred in both men and women, and resulted in an excess of ∼5 cases per 100 000 individuals. Mason *et al*. hypothesized that folic acid fortification was responsible for these increases in CRC rates but because of limitations in the datasets a causal relationship could not be established. Nevertheless, these observations are provocative and cannot be accounted for by changes in the use of colonoscopy or other screening procedures which showed later increases from 1999 to 2001 (Mason *et al*, 2007). It should be noted, however, that the temporary increase in CRC incidence between 1996 and 1998 occurred against a background of gradually declining incidence over the decade as a whole which may in part be accounted for by increasing population intakes of folate-rich foods and folic acid supplements.

## Conclusions and future directions

Recent evidence suggests that an individual who chooses to increase their folate intake with folic acid supplements may experience either beneficial or detrimental effects in terms of CRC risk depending on the existence of early neoplastic lesions in their colorectal epithelium. This relationship may be further influenced by their genotype, and their prior level of dietary folate intake. The net effect on CRC incidence of increased folate intake across the population remains unknown, and depends on whether the preventive influences against neoplastic transformation in the normal colorectal epithelium are out-weighed by later enhancement of the growth of early neoplastic lesions that may develop despite early protection. Thus the efficacy and safety of folate in CRC prevention remains undetermined and will depend on the relative size of these two potential effects, the level of increased exposure, and the nature of the population exposed in terms of age and other CRC risk factors. Only randomised primary preventive trials can provide definitive data to address these issues, and would ideally recruit participants of young age who are unlikely to have pre-existing early colorectal lesions. Although such studies will be logistically challenging and require a very significant investment of resources, the widespread voluntary usage of folic acid supplements coupled with mandatory folic acid fortification in some countries make clarification of both the efficacy and safety of these interventions a high priority. In the meantime, recommendations should be based on a synthesis of available data from observational data, genetic studies, mechanistic and animal studies, and randomised secondary preventive studies. These issues should be considered carefully by government authorities in the United Kingdom and other countries where mandatory fortification is currently being considered.

## Figures and Tables

**Table 1:** Details of published randomised double-blind placebo-controlled trials of folic acid for prevention of colorectal adenoma recurrence

							**Intervention**		
							**Placebo**	**Folic acid**	
**Study**	**Year^a^**	**Country**	**Folic acid dose (*μ*g/day)**	**Duration (years)**	***N* b**	**Outcome**	***N* outcome/ total (%)**	***N* outcome/ total (%)**	**RR (95%CI)^c^**
Paspatis *et al*	NS	Greece	1000	1	60	Adenoma incidence	11/29 (38)	7/31 (23)	0.48 (0.13–1.68)^d^
				2	60	Adenoma incidence	8/29 (28)	4/31 (13)	0.39 (0.08–1.72)^d^
Cole *et al*	1994–98	USA	1000	3	987	Any adenoma incidence	206/486 (42.4)	221/501 (44.1)	1.04 (0.90–1.20)
						Advanced adenoma incidence^e^	42/486 (8.6)	57/501 (11.4)	1.32 (0.90–1.92)
						No of adenomas 1–2	168/486 (34.6)	174/501 (34.7)	1.00 (0.85–1.19)
						⩾3	38/486 (7.8)	47/501 (9.4)	1.20 (0.80–1.81)
									
				6–8	607	Any adenoma incidence	113/304 (37.2)	127/303 (41.9)	1.13 (0.93–1.37)
						Advanced adenoma incidence^e^	21/304 (6.9)	35/303 (11.6)	1.67 (1.00–2.80)
						No of adenomas 1–2	100/304 (32.9)	97/303 (32.0)	0.97 (0.77–1.22)
						⩾3	13/304 (4.3)	30/303 (9.9)	2.32 (1.23–4.35)
Logan *et al*	1997–2001	UK	500	3	853	Any adenoma incidence	105/421 (24.9)	115/432 (26.6)	1.07 (0.85–1.34)
						Advanced adenoma incidence^e^	52/421 (12.4)	52/432 (12.0)	0.98 (0.68–1.40)

a Time period of participant recruitment.

b Number of subjects available for outcome assessment.

c Relative risk and 95% confidence interval.

d Calculated from published raw data.

e Advanced adenoma defined as adenomas ⩾1 cm in diameter, adenomas with villous or tubulovillous histology, adenomas with severe dysplasia, or diagnosis of colorectal cancer; NS, not stated.
